# Treatment of impacted or retained second molars with the miniscrew-supported pole technique: a prospective follow-up study

**DOI:** 10.1186/s40510-022-00432-5

**Published:** 2022-10-10

**Authors:** Carmen Lorente, Pedro Lorente, Maria Perez-Vela, Cristina Esquinas, Teresa Lorente

**Affiliations:** 1 Department of Human Anatomy and Histology, University of Zaragoza, Zaragoza, Spain; 2 Lorente Orthodontic Clinic, Paseo Constitución 29 (local), 50001 Zaragoza, Spain; 3 Valle de Hebrón Hospital Research Institute, Paseo del Valle Hebron, 119-129, 08035 Barcelona, Spain

**Keywords:** Retained/impacted molar, Ectopic eruption, Uprighting, Surgical procedure, Miniscrew, Skeletal anchorage, Pole technique, Molar angulation

## Abstract

**Background:**

Eruption disturbances of permanent molars are uncommon; however, it is important to treat them as soon as they are diagnosed. The main objective was to analyze the effectiveness of the “miniscrew-supported pole technique,” a surgically assisted orthodontic procedure to force the eruption of impacted/retained second molars (M2s) when there are indicators of complex molar inclusion. An observational prospective study was carried out during a 2-year period. Sociodemographic, clinical and low-dose scanner variables were taken at baseline (T0). Follow-up variables (T1) were the time between surgery and tooth eruption, radiographic measurements, debonding of buttons, failure rate of miniscrews and success rate of eruption.

**Results:**

A total of 21 patients (mean age of 13.9 years) with 24 retained/impacted M2s were recruited; 13 molars were maxillary (54.2%) and 11 (45.8%) were mandibular. Six (25%) were impacted molars and 18 (75%) primarily retained. At T0, molar angulation was mesial in six molars (25%), distal in five molars (20.8%) and 13 molars were vertically positioned (54.2%). Infraocclusion degree was moderate in four (16.7%) molars and severe in 20 (83.3%). Only three (12.5%) third molars were removed due to lack of space. All M2s managed to erupt, achieving a success rate of 100%; however, two molars of the same patient did not achieve occlusion. The period of eruption after surgery was 126.8 (117.3) days. Anatomical radicular alteration was the only variable independently related to a longer time of treatment (*p* = 0.027).

**Conclusions:**

The pole technique, using one mesial miniscrew and simple orthodontic mechanics, applies forces that succeed in erupting complicated retained/impacted M2s in a short period of time and with a low failure rate.

## Background

Failure of eruption of permanent molars is infrequent. Impacted molars are reported usually as occurring in the normal population with a prevalence of 0.01–4.3% in the case of the first permanent molar, and 0.06–2.3% in the second molar (M2) [1]. In most cases, the unerupted molars are mandibular M2s (65%), followed by maxillary M2s (21%) [2]. The condition is more common in females than in males, with a rate of 2.25:1 [3], and unilateral more frequent than bilateral [4].

The etiology of this type of eruptive alteration may be due to factors such as unusual orientation of the dental germ followed by an abnormal eruptive path; inadequate length or insufficient space for eruption; presence of supernumerary teeth or odontogenic neoformations such as tumors, follicular cysts, and odontomas; presence in the gingiva of scar tissue or fibromatous or hyperplastic alterations; root resorption of an adjacent tooth; delayed eruption of second premolars or presence of ankylosed deciduous molars; and idiopathic factors. According to the etiology, included M2s can be diagnosed as impacted (a tooth that is predicted to remain unerupted because of a physical barrier or deflection along its eruption path) or retained (eruption cessation of a normally placed and developed tooth germ for which no physical barrier can be identified) [5, 6].

It is important to treat this kind of molars as soon as they are diagnosed [7]. Unerupted molars can cause complications such as cysts, infection (pericoronitis, abscess), overeruption of the opposing teeth or adjacent tooth pathology like root resorption, caries and periodontal problems [1, 8]. The absence in the oral cavity of these permanent molars can lead to alterations of dental aesthetics, masticatory function, and dental arch stability, so it is an anomaly that must be solved [9]. Treatment options for deeply impacted molars include surgical extraction, surgical uprighting or repositioning of the tooth, and surgical uncovering with orthodontic-assisted forced eruption, being this last option the most conservative alternative [10]. Currently, surgical uncovering with skeletal anchorage for orthodontic management of the molar is being used as another option to treat impacted M2s, because it allows the application of a higher amount of force to the molar and avoids dental side effects with less complex biomechanics [7, 11–13]. However, most of the previously defined techniques employed miniplates or miniscrews distal to the included second molar, being difficult to apply these techniques in the maxilla and forcing to the extraction of the third molar.

Due to the low prevalence of ectopically erupted permanent molars, uniformity in the management of these teeth is lacking [1]. Many surgical and orthodontic solutions have been proposed by several authors [7–12]. During the past years, our group has applied a surgically assisted orthodontic procedure called the miniscrew-supported pole technique to force the eruption of impacted or retained molars regardless their angulation and location [14]. The hypothesis was that this technique allows to achieve eruption in short periods of treatment time, notwithstanding characteristics related to the inclusion M2. The purpose of this study was to evaluate the safety and effectiveness of this ortho-surgical technique.

## Methods

### Study design and study population

This is an observational, prospective study examining a cohort of patients diagnosed with impacted or retained M2s, using a cone beam computed tomography (CBCT) at a private orthodontic practice. Some patients, after these initial orthodontic records, underwent a panoramic radiograph during the follow-up, to control the eruption of the M2, avoiding a new CBCT to minimize the radiation. A total of 21 patients with 24 impacted or retained M2s were included in this analysis.

Several authors considered the following risk indicators when diagnosing included molars: (1) older than 14 years old [15]; (2) vertical, distal, or mesial M2 angulation ≥ 45° [16–18]; (3) severe bone depth of the M2 [5]; (4) proximity to the inferior alveolar nerve canal or the maxillary or mandibular cortical bone; (5) closed apex [5]; (6) alteration of the root apex; and (7) signs of primary M2 retention like increased dental follicle [15, 16]. We decided to use the pole technique in selected cases, when a previous conventional surgical exposure without the placement of a miniscrew was unsuccessful or when the patient presented at least three of the mentioned indications, since this technique, despite being more invasive, exerts more force than conventional surgery. The selection criteria are shown in Table 1.

**Table 1:** Selection criteria

Inclusion criteria
Subjects must provide written informed consent prior to performance of study, specific procedures or assessments and must be willing to comply with treatment and follow-up
Second molar impaction or primary retention (maxilla or mandible)
Initial records with CBCT
Surgery done with the mechanics of the miniscrew-supported pole technique. These patients must present at least three of the following indications:
Older than 14 years old
Vertical, distal, or mesial M2 angulation ≥ 45°
Severe bone depth of the M2
Proximity to the inferior alveolar nerve canal or the maxillary or mandibular cortical bone
Closed apex
Alteration of the root apex
Signs of primary M2 retention like increased dental follicle
Previously unsuccessful surgical exposure
Exclusion criteria
Patients with existing periodontal disease (patients with bleeding on probing, pocket depths > 3 mm and decreased bone diagnosed from baseline radiography)
Complicated medical or social history where surgery, orthodontic treatment or periodontal probing may be contraindicated and /or syndromic patients
Age > 25 years

### Surgical procedure of the miniscrew-supported pole technique

Patients included in this study underwent the miniscrew-supported pole technique described by Lorente et al. [14] as these molars presented at least three or more indicators of ectopic eruption (Table 1). After the mouth is rinsed with chlorhexidine digluconate 0.2% mouthwash, a 0.021 × 0.025-inch stainless steel buccal splint is placed on the three adjacent mesial teeth. A small step should be made on the stainless steel wire and placed between the first molar and second premolar to pass the pole. The splinting also reinforces the anchorage unit avoiding unwanted movement of the teeth as uprighting a M2 is notorious for causing the anchor teeth to move mesially or for the adjacent molar and premolars to be intruded [19].

**Fig. 1:**
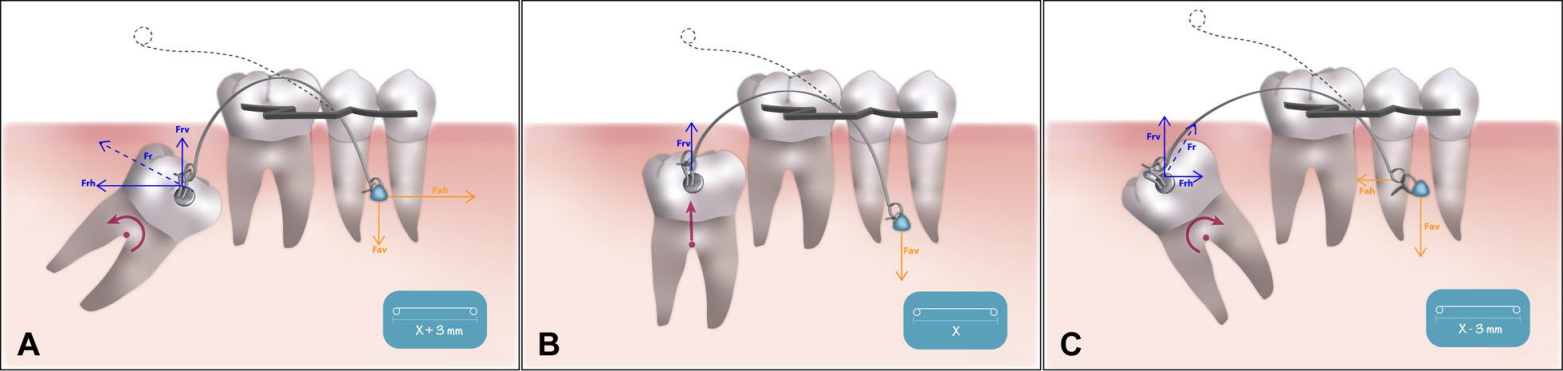
Selection of the pole length depending on the included molar inclination: **a** mesial. **b** vertical. **c** distal. Force delivered to the pole arm and moment acting on the impacted/retained molar (pink). *F*_r_ (blue), reciprocal force; *F*_rv_, vertical/extrusive component of *F*_r_; *F*_rh_, horizontal component of *F*_r_. *F*_a_ (orange), activation force; *F*_av_, vertical/extrusive component of *F*_a_; *F*_ah_, horizontal component of *F*_a_

**Fig. 2:**
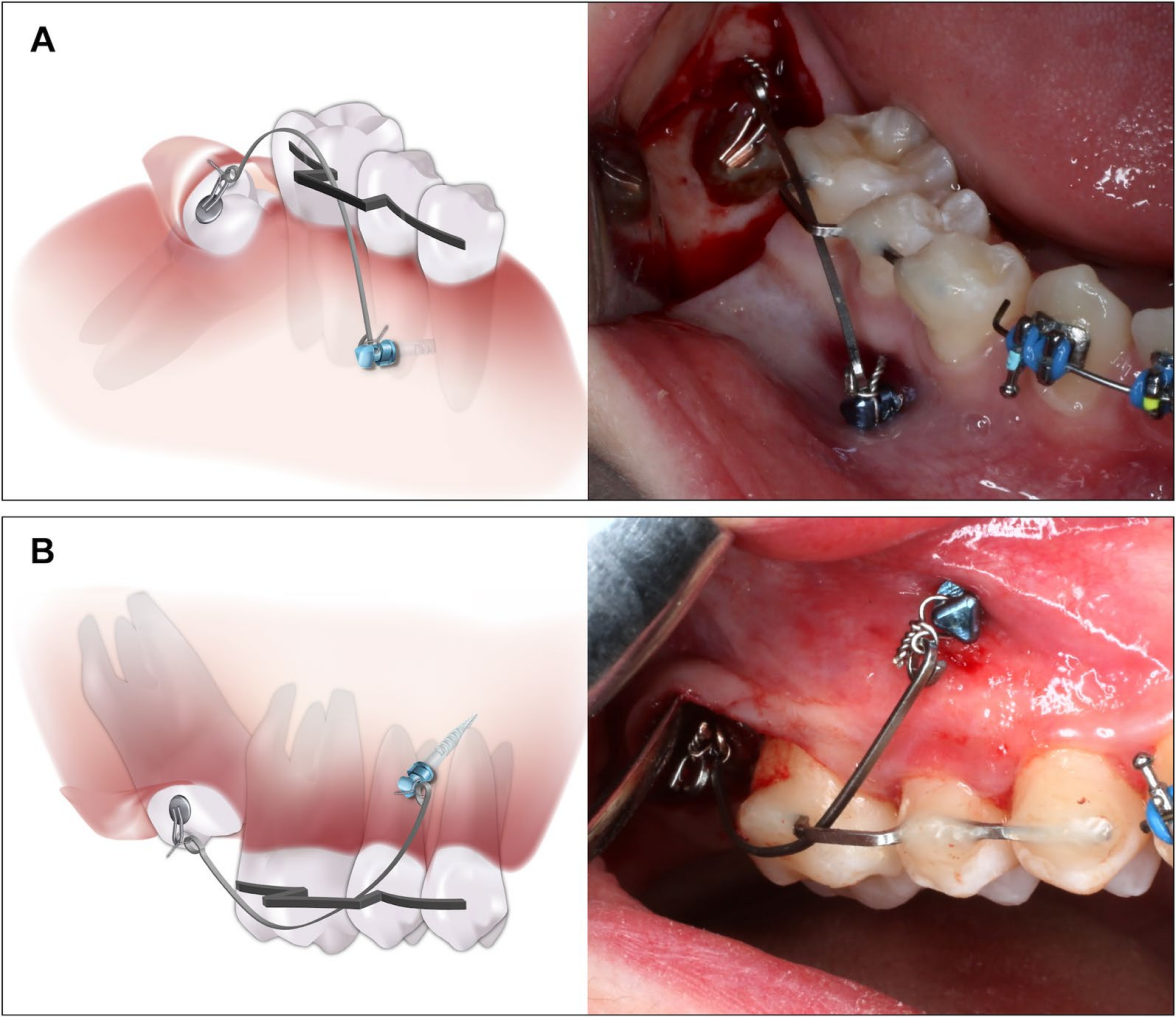
Diagram and intraoral photograph of the miniscrew-supported pole technique: **a** maxillary molar. **b** mandibular molar

While waiting for the local anesthesia to take effect, a loop should be made on one end of a 0.019 × 0.025-inch NiTi archwire. The shape memory effect of this archwire will provide the extrusive force to the tooth. Next, a miniscrew of 10–12 mm long and 2 mm in diameter is used as anchor of the pole (VectorTAS, trademark of Ormco Corporation, Orange, CA). When the molar is located in the mandible, the miniscrew is inserted into the gingiva between the first and second premolar at 90° to the cortical surface. On the other hand, if it is a maxillary molar, the miniscrew is inserted into the interradicular space between the first molar and second premolar at 5–11 mm from the alveolar crest and with an insertion of 30-45° to the dental axis to avoid root damage [20]. A mucoperiosteum flap is raised to expose the molar and a minimal amount of bone is removed to allow bonding of an orthodontic attachment. Next, the pole is measured. The previously made loop is placed on the miniscrew, making another helix at the other end of the wire according to the angulation of the molar. Three different pole lengths can be described depending on the point of force application in relation to the center el resistance: (1) if the molar has a vertical position, the pole length should be the distance between the bonded attachment and the miniscrew generating mainly an occlusally directed force; (2) if the molar has a mesial angulation, this length should be increased 3 mm generating a force with two components, an extrusive one and a distalizing one; and (3) if the molar has a distal angulation, conversely, this length should be decreased 3 mm generating a force with two components, an extrusive one and a mesializing one (Fig. 1) [14]. The pole should first be connected with the bonding attachment and then with the miniscrew, placing the pole through the previously made step. This connection will be made through a metallic ligature wire (0.012-in stainless steel). The activation process involves considerable compression of the segments. The higher the compression is, the higher the distalizing force will be. The gradually diminishing curvature of the segments during the deactivation process implies that the distribution of the resulting force components will alter throughout deactivation. The shape memory effect of the NiTi archwire will require the activation of the system only on the day of the surgery (Fig. 2). The generated force span on the molar is between 150 and 200 gr. However, the location of the center of resistance (CR) of the impacted/retained molar will be influenced by additional factors such as the amount of bonny embedding of the coronal third of the tooth, presence of follicular cysts, and contact with adjacent teeth or root anatomical alterations. For this reason, the moments and forces generated for each molar are independent and difficult to quantify.

The mucoperiosteum flap is then sutured into place, covering the impacted or retained tooth, and the sutures are removed after 10–14 days.

This technique can be applied on both maxilla and mandible arches. At our clinic, it has been used mainly for impacted or retained M2s. However, it has also been useful for canines, first molars and even third molars in selected cases with another tooth agenesis.

### Data collection and variables

Data were collected over a 2-year period (from May 2016 through May 2018). Orthodontic records including an initial CBCT were taken in all the patients. The end of the follow-up was December 2018.

Sociodemographic (age, gender) and clinical variables (extraction of third molars prior to surgery, performing a previous conventional surgery) and low-dose CBCT scanner measurements (molar angulation, molar bone depth, width of the dental follicle, proximity to the inferior alveolar dental nerve canal or maxillary/mandible cortical bone, closed apex or alteration at the apex of the root, indicators of molar retention) were collected at baseline (T0). The end of the follow-up in this study was defined as the moment in which the retained or impacted M2 broke out through the overlying mucosa (T1).

**Fig. 3:**
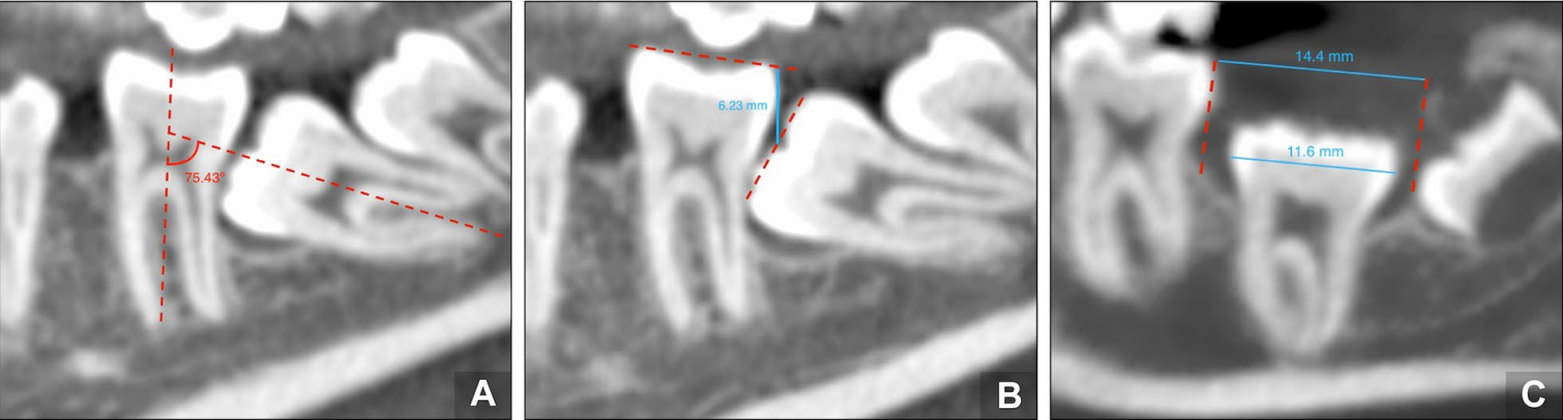
Scan measurements of the impacted/retained M2: **a** molar angulation. **b** degree of infraocclusion. **c** lack of space for eruption

For measuring the angulation, the angle formed between the middle axis of the adjacent tooth and the impacted or retained molar was taken [21]; it was considered mesial when the angle formed was greater than + 10°, vertical when the angulation was between + 10° and − 10°, and distal when it was less than − 10° (Fig. 3a).

The degree of infraocclusion [22] was also measured in millimeters (mm) from the occlusal plane to the midpoint of the occlusal surface of the impacted or retained molar and was classified as mild (when the midpoint of the occlusal surface of the molar was located in the area between the occlusal plane and the line passing through the contact point), moderate (in the area between the line passing through the point of contact to the cementoenamel junction [CEJ]), and severe (at the level of the CEJ or more apically) (Fig. 3b).

The anatomy of the roots of the included M2 was also evaluated, recording if the apex was opened/closed and the presence of anatomically radicular alteration such as dilacerations. The width of the dental follicle (mm) was measured as the largest distance from the crown of the molar to the periphery of the follicle. A result greater than 2 mm was considered an enlarged dental follicle [23].

In this study, molars were classified according to whether they were impacted or retained. In molars that were considered retained, the difference between the distance from the distal height of the contour of the first molar to the ramus or maxillary tuberosity parallel to the occlusal plane (or the mesial height of the contour of the third molar if present) minus the mesiodistal width of the mandibular M2 crown was measured to ensure that the delayed tooth eruption was not due to a lack of space (Fig. 3c) [24].

Follow-up variables were the time between surgery and the appearance of the tooth in the mouth (days), radiographic assessment of tooth position and angulation, debonding of buttons, failure rate of miniscrews and success rate of eruption. The molar eruption was considered successful if it erupted in a good vertical position with the occlusal surface < 2 mm from the occlusal plane [6]. The tooth was monitored at visits every two weeks. Close monitoring was recommended to prevent excessive extrusion of the molar because of the amount of force. When the molar erupts, the miniscrew is removed and brackets and tubes are placed on the premolars and molars to continue with dental alignment. The main outcomes were the rate of success of the technique defined as the appearance of the tooth in the oral cavity, and the time between the surgery and the molar eruption.

### Statistical analyses

Sample size was calculated according to the main outcome “rate of success.” Previous studies showed 90% success rate for orthodontic treatment method of maxillary impacted canines [25]. We estimated that the treatment of impacted or retained M2s with this technique could achieve higher success rate (98–100%). Using a 90% confidence interval, a total of 24 patients randomly selected would be necessary.

To describe the qualitative variables, absolute frequencies and percentages were used. The description of quantitative variables was performed using the mean, standard deviation (SD), median and interquartile range (IQR). The Kolmogorov–Smirnov test was used to assess the normality of distributions.

The main outcome was the time of treatment (days), and it was compared according to sociodemographic and clinical factors. In the case of categorical variables, the Mann–Whitney *U* test was used, and the Spearman correlation test was carried out to analyze linear relationships. The Chi-squared test (Fisher’s exact for frequencies < 5) was used to compare categorical variables.

To identify variables related to the time of treatment, a back stepwise linear regression model was developed. Variables with a significance of < 0.2 in the univariate analysis were included as independent variables. The results were described with a beta coefficient with a 95% confidence interval (CI) and *p*-values. For all the tests, *p* values < 0.05 were considered statistically significant. The software R Studio (v3.5.2) was used for the statistical analyses.

### Ethical issues

The study was carried out according to the principles of the Declaration of Helsinki and the prevailing norms for performing investigations in humans. Data confidentiality was ensured according to the Law of Data Protection 15/1999. The study was approved by the Clinical Research Ethics Committee, and all the participants provided signed informed consent.

## Results

### Study sample

A total of 48 molars were operated on during the study period, of which 28 molars met three or more indications to carry out the miniscrew-supported pole technique [14]. After applying the selection criteria, 21 patients with 24 impacted or retained M2s were included in this analysis. Patients presented a mean age of 13.9 (SD 1.9) years (range, 12–21 years) and 52.4% were male (*n* = 11). Thirteen teeth were maxillary (54.2%) and 11 were mandibular molars (45.8%). Six (25%) were impacted molars and 18 (75%) were primarily retained. No molars were secondarily retained teeth.

### Baseline CBCT variables

The distribution of molar angulation at T0 was mesial (*n* = 6; 25%), vertical (*n* = 13; 54.2%), and distal (*n* = 5; 20.8%). The mean angle of the long axis of the impacted or retained tooth to the adjacent tooth was 21.4 (SD 22.1) degrees (mesial 48.5 [SD 25.4], vertical 7.7 [SD 1.7], and distal 24.3 [SD 16.5]). According to the infraocclusion degree, the distribution of impacted or retained molars at T0 was mild (*n* = 0; 0%), moderate (*n* = 4; 16.7%) and severe (*n* = 20; 83.3%). The mean distance from the occlusal surface of the included tooth to the occlusal plane was 7.7 (SD 3) mm.

The anatomy of the molar was also evaluated, observing that 11 molars (45.8%) had a closed apex, and nine (37.5%) had an anatomically radicular alteration. In relation to the dental follicle, it was increased in 13 molars (54.2%).

The relationship that existed between the size of the follicle and the type of eruptive alteration (impaction or retention) was analyzed, but no differences were observed between retained and impacted teeth (2.1 [SD 1.4] vs 2.1 [SD 0.9], *p* = 0.916).

The adjacent third molar was not removed in 12 cases (50%), nine (37.5%) presented agenesis, and the remaining three (12.5%) were extracted due to lack of space (Table 2).

**Table 2:** Sample characteristics

Variable			Total (*N* = 24)
Age (years)		Mean (SD)	13.9 (1.9)
Gender	*n* (%)	M	14 (58.3%)
		V	10 (41.7%)
Impacted/retained molar	*n* (%)	17	5 (20.8%)
		27	8 (33.3%)
		37	3 (12.5%)
		47	8 (33.3%)
Molar location	*n* (%)	Inferior	11 (45.8%)
		Superior	13 (54.2%)
Angulation	*n* (%)	Vertical	13 (54.2%)
		Mesial	6 (25%)
		Distal	5 (20.8%)
Degree of molar angulation	Mean (SD)		21.4 (22.1)
	Median (IQR)		9.9 (7.4; 26.2)
Impaction depth	*n* (%)	Moderate	4 (16.7%)
		Severe	20 (83.3%)
Distance from the occlusal surface	Mean (SD)		7.7 (3)
	Median (IQR)		7.1 (5.5; 10.3)
Closed apex	*n* (%)		11 (45.8%)
Anatomical radicular alteration	*n* (%)		9 (37.5%)
Type of eruptive alteration	*n* (%)	Impaction	6 (25%)
		Retention	18 (75%)
Size of the dental follicle (mm)	Mean (SD)		2.1 (1)
	Median (IQR)		2.1 (1.3; 2.5)
Increased dental follicle	*n* (%)	Present	13 (54.2%)

*SD*, standard deviation; *IQR*, interquartile range

### Follow-up variables

The period of eruption after surgery was 126.8 (117.3) days (median of 99.5 [IQR: 48.3–133.5] days). No attachments were debonded from any molar; however, there was a miniscrew failure in one case, so it was replaced.

In this sample, two molars had been previously orthodontically-assisted with surgical exposure without success after a mean period of traction of 10 months. In these two teeth, the pole technique was carried out, obtaining molar eruption after a mean period of 4.5 months.

**Fig. 4:**
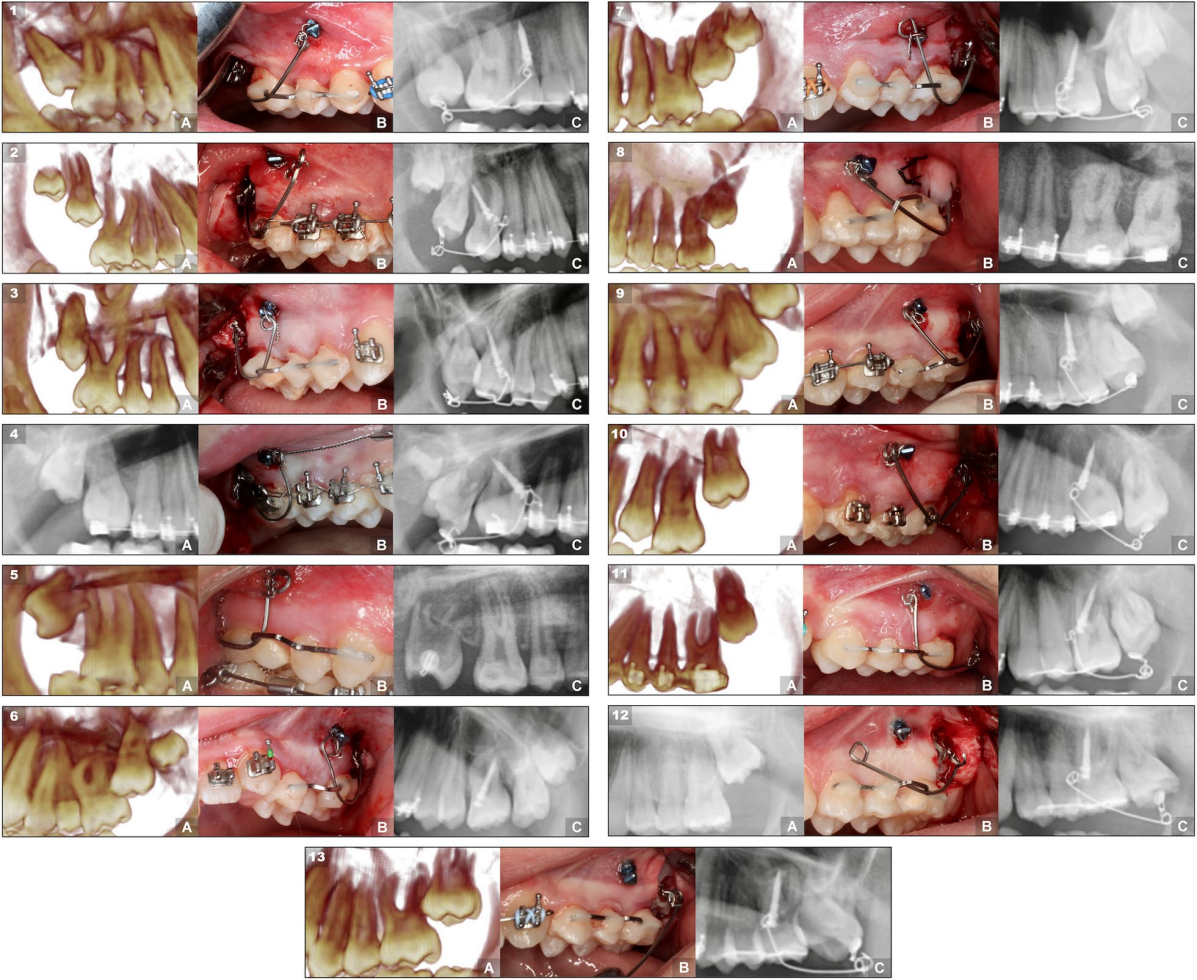
Case series of maxillary M2s treated with the miniscrew-supported pole technique. The 13 cases are organized according to their angulation, from mesial to distal, showing first the right ones and then the left ones. Each case is composed of three images that represent three moments during the treatment: **a** pretreatment radiograph showing the impacted or retained upper molar. **b** molar with the pole technique surgery carried out. **c** radiograph showing the final result with the molar in a correct position and angulation

**Fig. 5:**
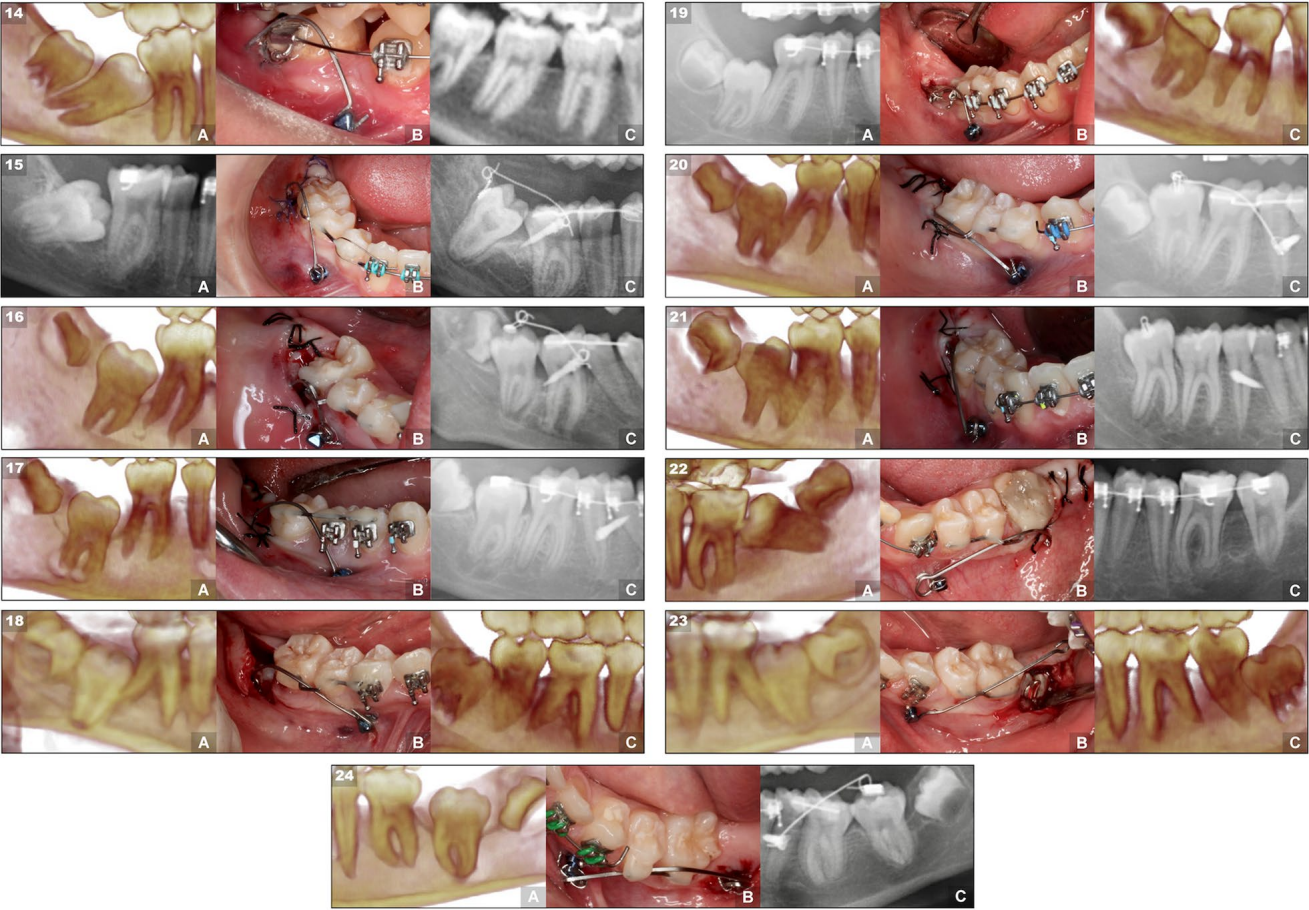
Case series of mandibular M2s treated with the miniscrew-supported pole technique. The 11 cases (from 14 to 24) are organized according to their angulation, from mesial to distal, showing first the rights and then the left ones. Each case is composed of three images that represent three moments during the treatment: **a** pretreatment radiograph showing the impacted or retained lower molar. **b** molar with the pole technique surgery carried out. **c** radiograph showing the final result with the molar in a correct position and angulation

All molars surgically operated on managed to erupt achieving a success rate of 100%. However, one molar did not reach the ideal occlusion despite appearing in the oral cavity (Figs. 4 and 5).

Multivariate analysis showed no relationship between clinical or CBCT variables and time from surgery to eruption. Only molars presenting anatomical radicular alteration were independently related to a longer time of treatment until eruption, compared to molars not presenting the alteration (152.3 vs 69.1 days, *p* = 0.025) (beta: 107, *p* = 0.027) (Table 3).

**Table 3:** Time between surgery and the appearance of the tooth in the mouth (days) according to sociodemographic and clinical variables. Bivariate and multivariate analysis

	Time from surgery to eruption (days) (mean (SD))	*p* value	Stepwise linear regression analysis Beta coefficient (CI 95%, *p* value)^1^
Age (years)*	Rho: 0.235	0.125	
Gender		0.578	
Male	135.4 (112.7)		
Female	120.6 (124.4)		
Molar site		0.198	
Inferior	138.3 (138.4)		
Superior	117.1 (101)		
Angulation		0.557	
Vertical	151.9 (142.6)		
Mesial	111 (96.6)		
Distal	80.4 (37.3)		
Eruptive alteration		0.665	
Impacted	111.0 (96.6)		
Retained	132.1 (125.6)		
Size of the dental follicle*	Rho: 0.173	0.420	
Degree of angulation	Rho: 0.049	0.820	
Vertical dental axis	Rho: 0.080	0.710	
Radicular alteration		0.025	ß: 107 (CI 95% 85 to 150, *p* = 0.027)
No	86.7 (69.1)		
Yes	193.7 (152.3)		

*Rho: Spearman coefficient

^1^Linear regression model including variables with a *p* value

## Discussion

The management of impacted or retained molars has been a challenge for orthodontists. When an impacted or retained permanent molar is diagnosed, various parameters must be considered, mainly the angulation [21, 26] and the infraocclusion degree of the impacted or retained molar, which can be analyzed by applying a modified version of the Pell and Gregory’s classification [22, 27]. Unfortunately, the lack of uniformity in these classification criteria generates a substantial difference among the main studies in this field, making it difficult to establish comparisons [1, 10]. In our study of 24 molars, six (25%) were mesially tilted, 13 (54.2%) were in a vertical position, and five (20.8%) presented a distal inclination. Eighteen were retained (75%). The infraocclusion degree was moderate in four (16.7%) and severe in twenty (83.3%) molars. This prevalence discrepancy with other studies could be the consequence of the strict selection criteria for applying the miniscrew-supported pole technique, with a lower prevalence of mesial M2s (only > 45°) and a higher frequency of severe infraocclusion degree.

Most of the published studies that treat impacted molars with skeletal anchorage have a time of traction that varies from 4 to 23 months [12, 28, 29]. Unfortunately, it is difficult to compare these data to the mean duration for eruption of 4.23 months obtained in our study, as most articles are case reports based on secondary retained molars with mesial inclination [5, 30].

In the multivariate analysis, we found that anatomical radicular alteration was the only variable independently related to longer time of treatment. When a root formation has an apical deviation from the normal axis of the tooth, it could generate a resistance in the eruption path, elongating the time until the molar erupts in the oral cavity.

According to the success rate after treatment, there are two remarkable studies. One recruited 135 retained or impacted M2s from three clinics, reporting a success rate of 42%. An orthodontic solution was given to 19 molars (14%), achieving a correct eruption only in eight [6]. The other one is a multicenter study with 170 patients with unerupted permanent molars, of which only 23.5% were orthodontically treated after surgical exposure, but unfortunately the success rate was not specified [1]. In our study, all 24 molars erupted successfully; however, two molars of the same patient did not achieve occlusion with the antagonist. No complication related to the procedure was evident, and only the failure of one miniscrew was reported, demonstrating the safety and effectiveness of the technique. It should be noted that since the average of the study was 14 years old, the risk of finding ankylosed M2 is lower, fact that would have produced the unintended adverse effect of dental intrusion of the teeth that support the pole. An initial low-dose CBCT and a panoramic radiograph prior to the removal of the device were taken to assess the molar position.

Many authors agree to perform treatment during adolescence, between 11 and 14 years of age [6, 9], before root formation is completed and before the third molar continues to develop above and on top of the M2 [10]. There is no clear consensus for the management of an impacted or retained molar. Extraction of a M2 is indicated when surgical exposure or orthodontic treatment cannot lead to eruption or a pathologic lesion is present [1]. Despite being the least conservative and successful, extraction of a M2, usually replaced by the third molar, is the most common treatment in approximately 60% of the cases [6]. The luxation of the tooth has an increased risk of complications, such as pulp necrosis, ankylosis, and root resorption [9, 10, 12]. Nowadays, an excellent option is the surgical uncovering with skeletal anchorage of the M2. Most of the described methods place the miniscrew distal to the molar [7, 8, 12, 28, 29]. However, they have some disadvantages, such as the miniscrew can be covered with soft tissue, the force span is shorter and these techniques usually cannot be applied in the maxillary arch. Finally, available space in the posterior mandibular arch is necessary, requiring the extraction of the third molar before the insertion of the miniscrew in the retromolar area [7, 8, 28, 29]. In our study, the extraction of the third molar was only prescribed if the bud was blocking the M2 eruption. From the 24 molars presented, only three cases needed to have the third molar removed. If the M2 does not erupt, it is always preferable to have the presence of the third molar.

The miniscrew-supported pole technique allows application of 150–200 g of force, because it uses a long lever arm that exerts great strength [11, 30]. The mesial location of the miniscrew is more comfortable for the patient, and it is a technique that can be used in both the maxilla and the mandible requiring the activation only the day of surgery [8]. The cantilevers anchored with mesial miniscrews that have been reviewed in the literature were designed to treat impacted mandibular molars with a mesial inclination, and which crowns were already erupted in the oral cavity, so the cantilevers had only an uprighting objective, and surgical exposition of the crown was not needed [11, 30]. However, in the technique presented in this case series, the molars are still embedded in the bone and completely covered by the gingiva, being impacted or retained either in mandible or maxilla, and depending on the inclination of the tooth, the cantilever can be modified to change the vectors of the force applied and generate a different effect on the impacted/retained molar.

The main limitation of the study is the reduced sample size, something otherwise expected considering the low prevalence of retained or impacted molars and that it was applied in selected molars. Another limitation is related to the design of the study, which ends at the moment of the eruption of the molar and does not take into account the buccolingual inclination of the molar in the initial CBCT data collection.

Every patient continues to be monitored to know the final result of the whole treatment and post-treatment follow-up, including CBCT images, periodontal measurements, and root resorption of the included molar and the adjacent teeth.

## Conclusion

The miniscrew-supported pole technique forces the eruption of molars with any type of angulation and location. Treatment of a case series presents good results at short-term and a high success rate. In addition, it seems comfortable to the patient and does not extend treatment time. The authors will continue monitoring these patients to report additional results.

## Data Availability

The data set supporting the conclusions of this article is included within the article. Further data sets are available from the corresponding author on reasonable request.

## Abbreviations

M2s: Second molars
CBCT: Cone beam computed tomography
NiTi: Niquel titanium
CR: Center of resistance
CEJ: Cementoenamel junction
SD: Standard deviation
IQR: Interquartile range
CI: Confidence interval
